# MITF and PAX3 Play Distinct Roles in Melanoma Cell Migration; Outline of a “Genetic Switch” Theory Involving MITF and PAX3 in Proliferative and Invasive Phenotypes of Melanoma

**DOI:** 10.3389/fonc.2013.00229

**Published:** 2013-09-11

**Authors:** Michael R. Eccles, Shujie He, Antonio Ahn, Lynn J. Slobbe, Aaron R. Jeffs, Han-Seung Yoon, Bruce C. Baguley

**Affiliations:** ^1^Department of Pathology, University of Otago, Dunedin, New Zealand; ^2^Malaghan Institute of Medical Research, Wellington, New Zealand; ^3^Auckland Cancer Society Research Centre, The University of Auckland, Auckland, New Zealand

**Keywords:** melanoma, phenotype switching, paired box transcription factors, microphthalmia-associated transcription factor, migration and invasion, pax3

## Abstract

Melanoma is a very aggressive neoplasm with a propensity to undergo progression and invasion early in its evolution. The molecular pathways underpinning invasion in melanoma are now just beginning to be elucidated, but a clear understanding of the transition from non-invasive to invasive melanoma cells remains elusive. Microphthalmia-associated transcription factor (MITF), is thought to be a central player in melanoma biology, and it controls many aspects of the phenotypic expression of the melanocytic lineage. However, recently the paired box transcription factor PAX3 was shown to transcriptionally activate POU3F2/BRN2, leading to direct repression of MITF expression. Here we present a theory to explain melanoma phenotype switching and discuss the predictions that this theory makes. One prediction is that independent and opposing roles for MITF and PAX3 in melanoma would be expected, and we present empirical evidence supporting this: in melanoma tissues PAX3 expression occurs independently of MITF, and PAX3 does not play a key role in melanoma cell proliferation. Furthermore, we show that knockdown of PAX3 inhibits cell migration in a group of “lower MITF” melanoma cell lines, while knockdown of MITF promotes cell migration in a complementary “higher MITF” group of melanoma cell lines. Moreover, the morphological effects of knocking down PAX3 versus MITF in melanoma cells were found to differ. While these data support the notion of independent roles for MITF and PAX3, additional experiments are required to provide robust examination of the proposed genetic switch theory. Only upon clear delineation of the mechanisms associated with progression and invasion of melanoma cells will successful treatments for invasive melanoma be developed.

## Introduction

Melanoma is a malignant neoplasm of the neural crest-derived melanocytes, the pigment-producing cells. Approximately 65% of cutaneous melanomas are thought to arise from individual cutaneous melanocytes, while ∼25% arise from a pre-existing nevus. The remaining melanomas (4–12%) appear to arise *de novo* with no identifiable primary tumor. Melanoma is a very aggressive neoplasm with a high risk of metastasis early in tumorigenesis. Despite numerous studies, the mechanisms underlying metastasis are complex, and a clear understanding remains elusive.

Acquisition of the ability of tumor cells to migrate represents a defining characteristic of cancer metastasis. However, cell migration is also necessary during embryogenesis and homeostasis of multicellular organisms. Indeed, recent studies suggest that melanoma cells revert to an embryonic program of gene expression involved in neural crest cell migration to support developmental plasticity and metastasis (1). Numerous factors are involved in the differentiation of melanocytes, and also in the control of cell migration.

*PAX3*, a member of the paired box family of genes, is a key developmental regulator of the neural crest and its derivatives, including melanocyte progenitors (2). PAX3 is expressed in melanoma tissues and cell lines, melanocyte cell lines (3, 4), and circulating melanoma cells. Several groups (5, 6) have shown that PAX3 protein is expressed in normal skin melanocytes and melanocytic lesions. *PAX3* expression is thought to contribute to cell survival and growth (3, 4) in the melanocytic lineage. Several studies have suggested that PAX3 expression is important in regulating the transition from an early melanoblast derived from the neural crest into mature melanocytes. Knockdown of PAX3 expression in melanoma cells leads to reduced or arrested cell growth, and the induction of apoptosis and/or senescence (3, 4).

Microphthalmia-associated transcription factor (MITF) is another important developmental regulator of neural crest and its derivatives (7). MITF has been suggested to be an important melanoma growth and survival factor (8). For instance, FOXD3, a neural crest-associated transcription factor, is able to repress MITF through non-canonical mechanisms, and regulate the lineage commitment of bi-functional neural crest-derived glial/melanocyte precursor cells into either the melanocyte or glial lineages (9). Analysis of MITF expression in melanoma cell lines, as well as melanoma tissues reveals marked variability in expression level, with some melanoma cell lines expressing up to 10-fold higher levels of MITFm, a melanocyte-specific isoform of MITF, than in other melanoma cell lines (10).

The variable levels of MITF expression in melanoma may have important consequences. Low levels of MITF expression have been shown to identify a small group of melanoma patients with high mortality. Agnarsdottir and colleagues showed that patients with melanomas where 25–75% of tumor cells stained with weak intensity for MITF expression using an anti-MITF antibody were at higher risk of death than patients with an overall strong MITF staining intensity (11). This effect of low MITF expression level on patient survival may be through various roles that MITF is thought to play in cell invasion- and proliferation-associated pathways. High MITF levels are thought to promote cell proliferation through the direct activation of the *DIAPH1* gene, one of many MITF target genes (12). High MITF expression has also been shown to transcriptionally activate microRNA *miR-211* expression, expressed from within the MITF target gene, *TRPM1*, which results in reduction in the levels of *POU3F2* (*BRN2*) mRNA (13). In contrast, under conditions of low MITF expression there is increased ROCK activity downstream of Rho, an important mediator of cell migration (12).

High expression of POU3F2 in melanoma represseses *MITF* expression (14). Moreover, POU3F2 both transcriptionally activates (including transactivation of *PAX3*) and represses genes leading to enhanced cell migration/invasion and stem cell-like characteristics (15–20). Indeed, POU3F2 is part of the phosphatidylinositol 3-kinase (PI3K)-PAX3-POU3F2 (BRN2) axis that has been proposed to promote melanoma cell invasion (21). Bonvin and colleagues showed that inhibiting the PI3K pathway causes down-regulation of POU3F2 and PAX3 expression, and that PAX3 directly bound to and transactivated the *POU3F2* promoter, up-regulating *POU3F2* expression. These findings implicate PI3K signaling in PAX3-dependent enhancement of *POU3F2* expression and melanoma cell invasion, while simultaneously inhibiting *MITF* expression (21).

A second signaling pathway that leads to cell migration also involves the downstream activation of PAX3 expression; fibroblast growth factor 2 stimulates STAT3-mediated regulation of *PAX3* expression in melanocytes (22). Moreover, STAT3 activation promotes cell migration through phosphorylation of STAT3, requiring Rho Kinase (ROCK) and JAK activity (23). Phosphorylated STAT3 transcriptionally activates *PAX3* expression in melanocytes, and the silencing of *STAT3* or *PAX3* using RNAi was recently shown to inhibit the growth of melanoma cells, particularly in melanoma cells that have acquired resistance to the BRAF inhibitor, vemurafenib (24). These studies suggest that PAX3 expression can promote melanoma progression, and that PAX3 plays an important role in acquired resistance and recurrence of melanoma following treatment with tyrosine kinase inhibitors.

Over the last 20 years a unique series of cell lines (NZM cell lines) from metastatic melanomas (MMs) occurring in New Zealand patients has been developed (25). To date the NZM cell lines have been characterized for cell cycle time, drug sensitivity, and driver gene mutation status. We recently profiled global gene expression in a panel of 25 of these cell lines, and showed that NZM and other melanoma cell lines could be classified into two major groups represented by relatively lower (6/25) or higher (19/25) *MITF* transcript levels. In the gene expression signature that distinguished the two groups there were 96 differentially expressed genes, many of which are known targets of MITF, which differed in expression in a similar fashion to MITF (26). The lower MITF cell lines were characterized as having higher rates of migration than higher MITF cell lines in Boyden chamber transwell assays and scratch assays (26).

Here we extend a hypothesis that we previously suggested; that PAX3 and MITF play independent roles in melanoma progression (10). Moreover, we showed previously that PAX3 does not regulate *MITF* in melanoma cells (10) and we now propose a “genetic switch” theory to explain phenotype switching (27), whereby a PAX3-POU3F2 axis and a MITF-*miR-211* axis function to negatively regulate each other. This predicts that PAX3 and MITF play distinct roles in signaling pathways that promote melanoma progression, and also predicts additional features expected in melanoma cells undergoing phenotype switching.

We present here several lines of experimental evidence supporting the notion that PAX3 and MITF expression indeed play independent roles in melanoma progression and cell migration. Firstly, we show that in melanoma tissues expression of MITF and PAX3 occur independently, and are variable from region to region, and furthermore that the expression of PAX3 is not correlated with Ki67 expression, a marker of cell proliferation. In addition, we show that in melanoma cell lines with lower levels of MITF expression, knockdown of *PAX3* expression inhibits melanoma cell migration, whereas in melanoma cell lines with higher levels of MITF, knockdown of *MITF* enhances cell migration. In addition we show that PAX3 promotes increased *POU3F2* transcript levels, which then leads to repression of *MITF* transcript levels. Lastly, we show that the morphological effects of knocking down *PAX3* versus *MITF* in melanoma cells differ. Taken together with earlier published data (10), the evidence suggests that there are distinct roles for PAX3 and MITF in melanoma progression and melanoma cell migration, thus providing supporting evidence for one of the key predictions of the genetic switch theory.

## Outline of a Genetic Switch Theory for the Involvement of PAX3 and MITF Axes during “Phenotype Switching” in Melanoma

**Figure 1:**
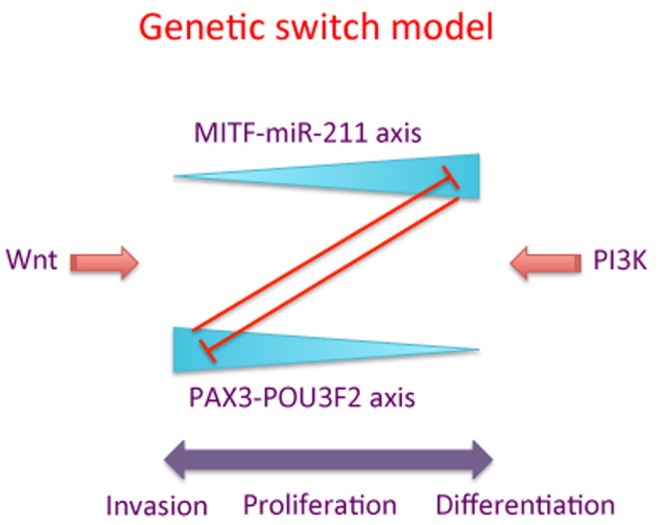
**A schematic of the “genetic switch” model**. The MITF-miR-211 axis is counterbalanced by the PAX3-POU3F2 axis, with high levels of each pathway inhibiting the corresponding other pathway. “Wnt” and “PI3K” represent one of several possible signaling pathways promoting the activity of MITF-miR-211 axis versus the PAX3-POU3F2 axis, respectively. High levels of the PAX3-POU3F2 axis (represented by the thick end of the wedge) are associated with cell migration and invasion of melanoma cells, while high levels of the MITF-miR-211 axis are associated with proliferation and/or differentiation of melanoma cells.

The genetic switch theory is outlined in Figure 1 and embodies the concept of two interacting signaling pathways represented by PAX3-POU3F2 and MITF-*miR-211*, two key pathways contributing to the strength of lineage commitment and phenotypic choice of individual melanoma cells.

In the genetic switch model we firstly note that PAX3 does not transcriptionally activate *MITF* in melanoma cells. Our earlier studies (10) support this notion. While in normal cells of the melanocytic lineage during melanocyte development PAX3 transcriptionally activates *MITF*, it is clear there is a difference in melanoma cells (10), and we propose this difference might be an important feature underlying the malignant potential of melanoma cells.

Our theory is consistent with data presented by Carreira and colleagues (12), who proposed that MITF functions like a “rheostat” with respect to cell migration. In their paper they suggested that, depending on the expression level, MITF plays a role in stem cell-like properties, and proliferation of melanocytes and melanoma cells, with an effect on terminal differentiation or senescence of cells at very high levels. Our genetic switch theory extends this model, and suggests that the rheostat model may only be half the story. In the genetic switch model we propose that the MITF-*miR-211* axis inhibits cell migration and promotes cell differentiation in cells where the relative expression of MITF is high. Conversely, we propose that when MITF levels are low, the expression of the PAX3-POU3F2 axis is high, and that this then promotes cell migration and stem cell-like properties (Figure 1).

The patterns of gene expression that we, and others, have previously characterized in melanoma cell lines (26–28) might reflect the bi-modal nature that would be predicted by the genetic switch model. In the NZM cell lines high expression of MITF and many of its target genes, and the low expression of another set of genes, were found to be typical of one gene expression signature, while the low expression of MITF and its target genes, and the high expression of the other gene set were typical of an alternative gene expression signature. Evidence of *in vivo* switching between two such alternative gene expression signatures has been suggested (27). Moreover, expression of the “lower MITF” gene signature corresponds to melanoma cells with a higher rate of migration, and migration rates in the “higher MITF” melanoma cell type were able to be enhanced by knocking down expression of *MITF* (26).

Within melanoma tissues, depending on localized exposure to external signals or cues, signals such as PI3K or STAT3 in the external “milieu” could activate the PAX3-POU3F2 axis, and therefore initiate migratory stem cell-like properties in melanoma cells (21, 22). Alternatively, external Wnt signals (for instance) might activate the MITF-*miR-211* axis, and so promote the expression of adhesion molecules to anchor migrating melanoma cells in order to colonize and proliferate in distal sites (8). Given predictions that relatively high numbers of stem-like cells may exist in melanoma, it may be that, *in vivo*, there is a relatively high frequency of the conversion rate from the proliferative phenotype to the migratory “stem cell-like” phenotype in melanoma cells compared to the reverse conversion rate.

Furthermore, accumulating evidence supports two models of how melanoma cells move, a cytoskeletal model of dynamic actin microfilaments, and a membrane flow model of plasma membrane deposition and recycling (23). In the former of these models it has been shown that STAT3 signaling plays an important role, which again provides supporting evidence for the role of a PAX3-POU3F2 axis in promoting cell migration.

Aside from what we have discussed above, several predictions arise from the proposed genetic switch theory. The first of these is that MITF and PAX3 should both have independent roles and expression patterns in melanoma cells. The second prediction is that the MITF-*miR-211* axis will prevail precisely when the PAX3-POU3F2 axis wanes, and vice versa. This prediction will need to be investigated in *in vitro* and *in vivo* models. *In vitro*, it is predicted that *MITF* and/or *miR-211* expression would be enhanced in melanoma cells with knockdown of the *PAX3*-*POU3F2* axis, and that *PAX3* and/or *POU3F2* expression will be enhanced in melanoma with knockdown of *MITF-miR-211* axis. We have already obtained preliminary evidence that the knockdown of *MITF* leads to increased *POU3F2* mRNA levels in NZM12 melanoma cells (He, Jeffs et al., unpublished data). The third prediction is that during periods of enhanced cell migration, melanoma cells would be under the influence of the PAX3-POU3F2 axis, and that melanoma cells not enhanced in migration would be under the influence of the MITF-*miR-211* axis. The fourth prediction is that high activity of the PAX3-POU3F2 axis would lead to stem cell-like features, with reduced pigmentation, reduced mitotic activity of melanoma cells, and enhanced resistance to drugs that inhibit proliferation, while high activity of the MITF-*miR-211* axis would lead to more strongly differentiated melanocytic features, enhanced pigmentation, and enhanced mitotic activity, with reduced resistance to drugs that inhibit proliferation. A fifth prediction that perhaps arises from all the above predictions, but is important nevertheless in translating to patients, is that the use of treatments in patients targeting MITF might result in enhancement of melanoma metastasis.

## Results

### PAX3 and MITF expression vary in their relative intensity in different regions of melanoma

**Figure 2:**
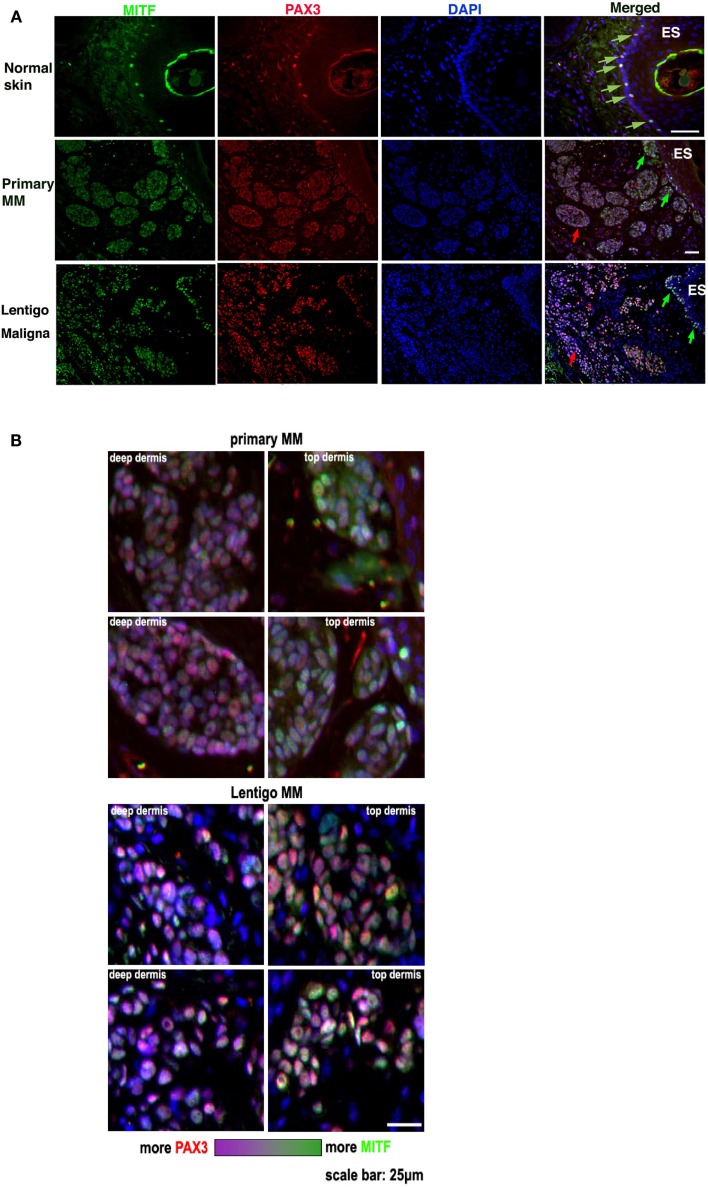
**PAX3 and MITF expression vary in their relative intensity in different regions of melanoma**. **(A)** The figure shows photomicrographs of dual immunofluorescent staining of MITF expression (green label), PAX3 expression (red label), DAPI nuclear staining (blue label), and a merged image in normal skin, metastatic melanoma (Primary MM), and Lentigo maligna melanoma. The scale bar, which varies in length on the images, represents 50 μm. **(B)** Higher magnification photomicrographs taken from the merged image in **(A)** of the Primary and Lentigo maligna melanomas show in greater detail the difference in the relative immunofluorescence color intensity of MITF labeling versus PAX3 labeling in the tumor cells immediately below the skin surface (“Top dermis”) versus cells located deeper in the tumor (“Deep dermis”). Below the panels is shown a color intensity scale, with one end representing relatively strong MITF intensity, and the opposite end representing relatively strong PAX3 intensity. The scale bar in the bottom right image is for all the panels and represents 25 μm.

Several experimental approaches were used to investigate whether PAX3 and MITF expression and function were independent in melanoma cells and tissues. In the first approach we used dual label immunofluorescence to compare the relative expression of PAX3 and MITF in adjacent regions within the same melanoma tissue section. To do this PAX3 and MITF were immunolabeled with different fluorophores, and the captured immunofluorescent images merged. The relative saturation (intensity) of the signals in the merged image were then visualized (Figure 2). Relative levels of MITF expression in normal skin melanocytes (observed as a single layer of cells in the basal layer of the epidermis – see Figure 2) seemed to vary in relation to each other more than the variation in PAX3 expression (Figures 2A,B). In melanoma the expression of MITF appeared to be generally more intense relative to PAX3, and tended to involve cells immediately underlying the epidermal surface (“Top dermis,” Figure 2B). Cells that expressed lower levels of MITF relative to PAX3 were often located deeper below the epidermal surface of the tissue (“Deep dermis,” Figure 2B). This was also observed in dysplastic nevi (not shown). We show two representative melanoma tissues (MM and lentigo maligna melanoma) where cells more distal to the epidermal surface showed a lower intensity of MITF expression relative to PAX3 expression, and a change in color saturation was observed in melanoma cells more distal to the epidermal surface compared to cells immediately below the epidermal surface (Figures 2A,B). These data suggest that variations in the relative levels of *PAX3* and *MITF* protein occur from region to region in melanoma tissues. Such variations in the expression of these factors could impact on the invasive behavior of melanoma cells.

### PAX3 is relatively infrequently co-expressed with Ki67 in melanoma tissue

**Figure 3:**
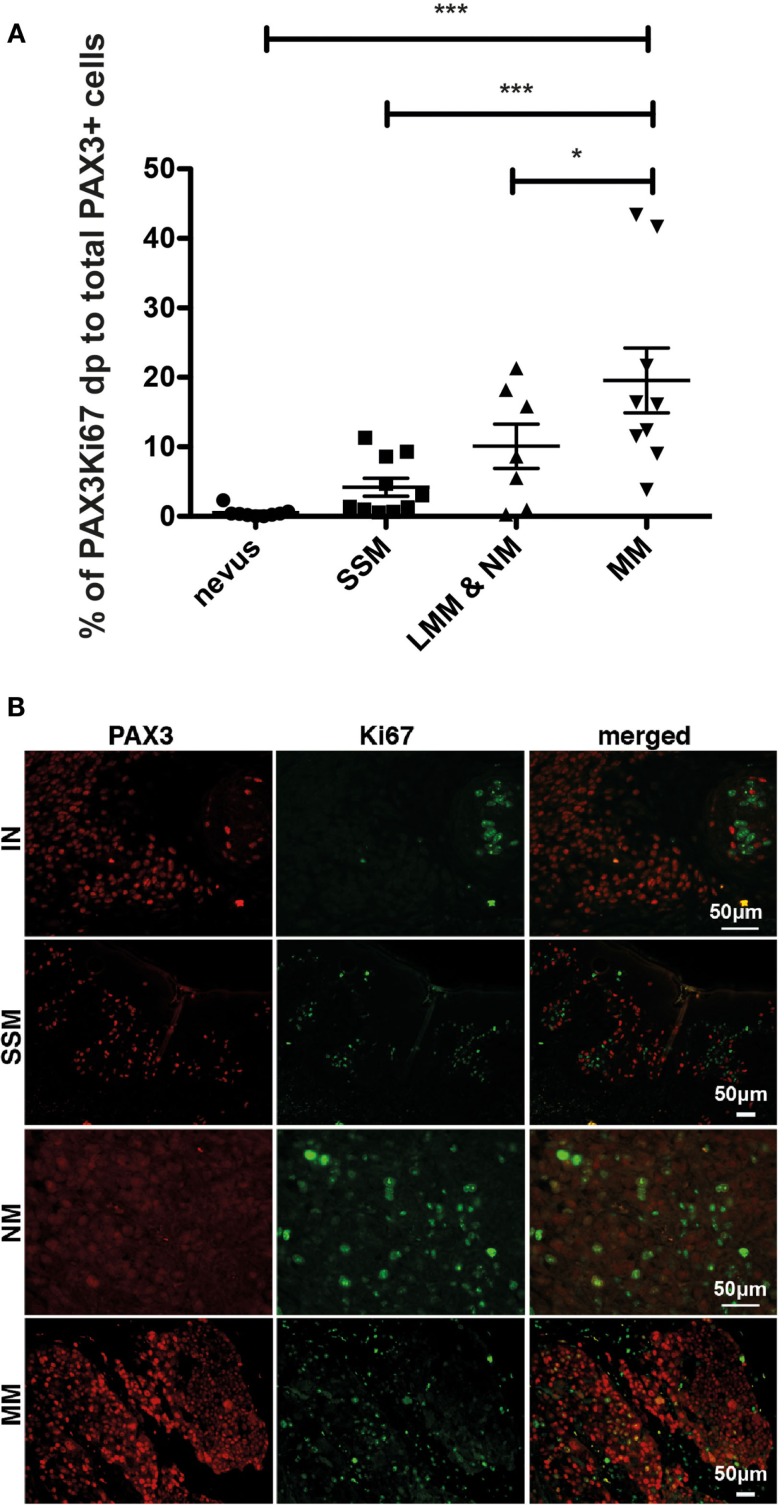
**Dual immunofluorescent staining of Ki67 and PAX3 in benign and malignant melanocytic lesions**. **(A)** Graph showing the percentage of PAX3 and Ki67 double positive (dp) cells as a percentage of the total number of PAX3-positive cells for superficial spreading melanoma (SSM), lentigo maligna melanoma and nodular melanoma (LMM & NM), and metastatic melanoma (MM), ***p < 0.001. **(B)** Photomicrographs showing the results of immunofluorescent staining for PAX3, Ki67, and merged images for an intradermal nevus (IN), superficial spreading melanoma (SSM), nodular melanoma (NM), and metastatic melanoma (MM). The scale bar, which varies in length on the images, represents 50 μm.

We next investigated whether expression of PAX3 is associated with loss of growth control in melanoma, which is a role that MITF has been implicated (8), as might be expected if PAX3 and MITF were to function in the same or similar pathways. We investigated whether PAX3 expression is co-localized with the cell proliferation marker, Ki67, scoring cells that were positive in immunofluorescence for both PAX3 and Ki67 as a percentage of the total number of PAX3-positive cells. Expression of PAX3 practically never co-localized with Ki67 expression in nevi (Figure 3), suggesting that the expression of PAX3 was in general not associated with proliferation in nevi. Co-localization of Ki67 with PAX3 was also relatively infrequent in melanomas including superficial spreading, lentigo maligna melanoma and nodular melanoma, and MMs, with an average of only ∼20% of cells co-expressing Ki67 and PAX3 in the latter (Figure 3). The observation that the expression of PAX3 does not markedly overlap with Ki67-positive melanoma cells (as the majority of PAX3-positive cells were Ki67-negative) suggests that PAX3 expression is not associated with cell proliferation in melanoma. The observed low frequency (∼20%) of incidental co-expression of PAX3 and Ki67 could simply reflect progressive deregulation of growth control in melanoma cells, as marked by Ki67 expression.

### RNAi-mediated knockdown of PAX3 gene expression leads to reduced POU3F2 mRNA levels and migratory behavior and increased MITF mRNA levels in melanoma cell lines

**Figure 4:**
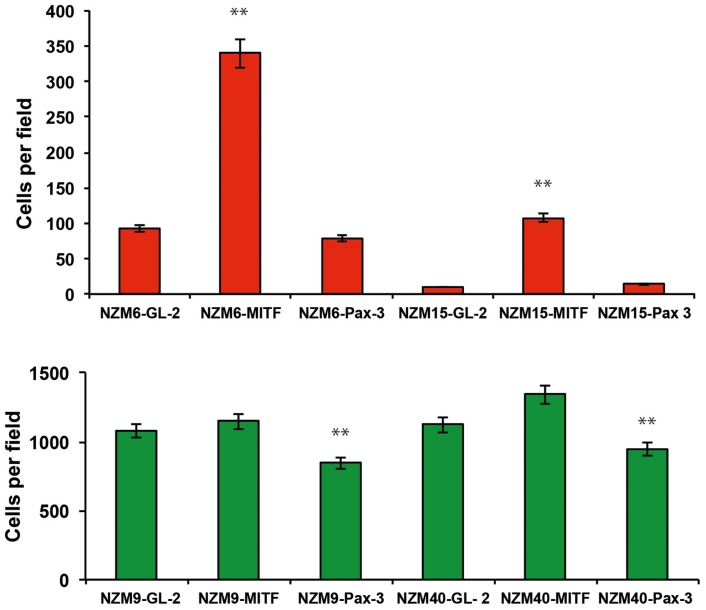
**Knockdown of PAX3 or MITF results in differential effects on the migration of melanoma cell lines *in vitro***. siRNA-mediated knockdown of *PAX3* inhibits the migration of melanoma cells expressing lower levels of MITF (green bars), but not melanoma cells expressing higher levels of MITF (red bars). Conversely, siRNA-mediated knockdown of *MITF* does not enhance the migration of melanoma cells expressing lower levels of MITF (green bars), but does enhance the migration of melanoma cells expressing high levels of MITF (red bars). **p < 0.001.

We previously characterized a panel of melanoma cell lines for expression levels of MITF and PAX3, and cell migratory behaviors (10, 26). Four melanoma cell lines chosen from this panel were transfected with siRNAs against *PAX3* to determine whether migration of the melanoma cells depended on *PAX3* expression. Knockdown of *PAX3* in NZM9 and NZM40, characterized to have lower levels of MITF expression and a high cell migration potential (10, 26), led to a significant decrease in migration rate (*p* < 0.001, Figure 4), whereas the knockdown of *PAX3* in NZM6 and NZM15 (characterized to have higher levels of MITF expression and a low migration potential) did not cause a significant change in the migration rates in transwell assays. In contrast, knockdown of *MITF* in NZM6 and NZM15 cell lines caused an average of ∼4-fold increase in migration rate in the transwell assays (*p* < 0.001, Figure 4), whereas in the highly motile NZM9 and NZM40 cell lines there was no significant change in migration potential observed with *MITF* knockdown (Figure 4).

**Figure 5:**
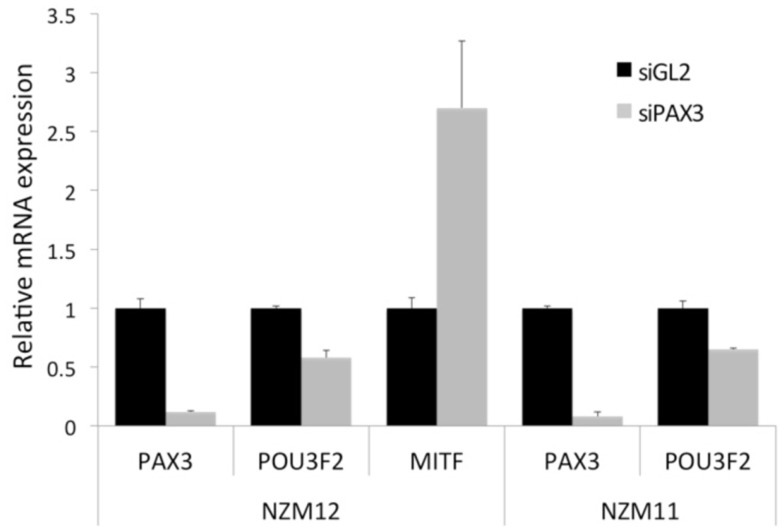
**Knockdown of PAX3 results in a decrease of *POU3F2* and an increase of *MITF* gene transcript levels in melanoma cell lines *in vitro***. Transcript levels of *PAX3*, *POU3F2*, and *MITF* mRNA were examined by RT-qPCR in NZM12, and in NZM11 (*PAX3* and *POU3F2* only) melanoma cell lines following RNAi treatment of the cell lines with either siRNAs against luciferase (siGL2) as a negative control, or against PAX3 (siPAX3). The results were calculated as the fold difference in transcript level relative to the level of the housekeeping gene *GNB2L1*, normalized to the siGL2 data.

In two different NZM melanoma cell lines (NZM11 and NZM12), one of which has previously been characterized to be a “lower MITF” cell line (NZM11), and the other cell line previously characterized as a “higher MITF” cell line (NZM12) (10, 26), the knockdown of *PAX3* expression resulted in decreased levels of *POU3F2* transcripts in both cell lines (Figure 5). In the NZM12 cell line there was a concomitant increase in *MITF* transcript levels, consistent with the proposed genetic switch hypothesis (Figure 5). An increase in the levels of both *MITF* mRNA and protein in NZM12, NZM11, NZM9, and NZM15 cell lines in response to PAX3 knockdown has also previously been reported (10). Interestingly, in two other “lower MITF” expressing melanoma cell lines (i.e., NZM9 and NZM40) there were undetectable levels of *POU3F2* expression. It is possible that these “lower MITF” melanoma cell lines have an alternative pathway to suppress *MITF* that does not involve *POU3F2* expression.

These data suggest that relatively higher expression of *PAX3* compared to *MITF* in “lower MITF” cell lines may facilitate cell migration in those melanoma cell lines, while relatively higher expression of *MITF* to *PAX3* in “higher MITF” cell lines may inhibit cell migration.

### RNAi-mediated knockdown of PAX3 or MITF gene expression leads to different phenotypic morphologies in melanoma cells

**Figure 6:**
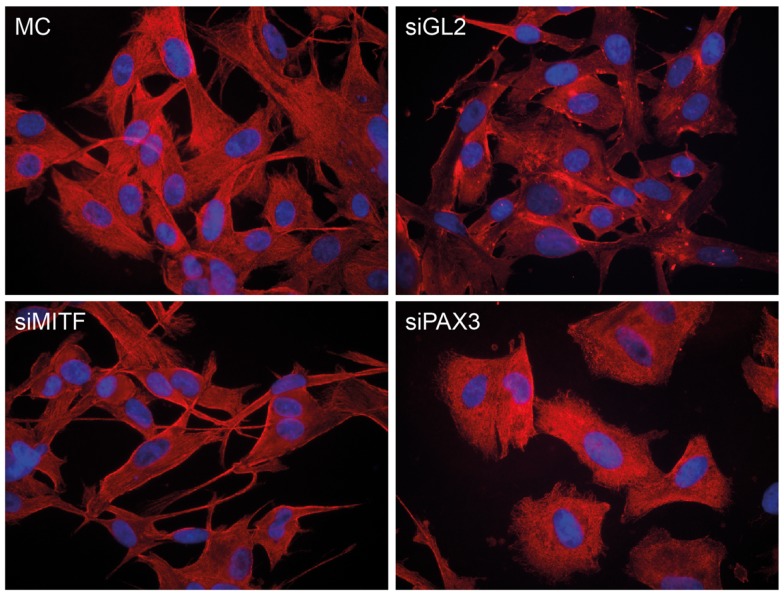
**Knockdown of PAX3 or MITF results in differential morphological effects in NZM15 melanoma cells**. NZM15 melanoma cells were grown in media without any transfection (MC), or transfected with siRNA to *luciferase* (siGL2) as a non-targeting control, siRNA to MITF (siMITF), or siRNA to *PAX3* (siPAX3) and then stained after 48 h with β-tubulin antibody.

The knockdown of either PAX3 or MITF expression had different effects on the morphology of NZM15 cells in culture (Figure 6). Knockdown of MITF in NZM15 cells led to cells with a fibroblast-like spindle-cell phenotype with dendrites protruding from the cells. In contrast, knockdown of PAX3 led to the cells acquiring an epithelial-like rounded phenotype with few dendrites.

## Discussion

The data presented here are consistent with and extend our previous work, in which we showed that melanoma cell lines with low levels of MITF expression (NZM9, NZM11, NZM22, NZM40, and NZM52) have higher (∼23-fold) migratory potential than melanoma cell lines with high MITF expression levels (NZM6, NZM12, NZM15, NZM42, NZM45). The latter cell lines have a low migratory potential using scratch and transwell (Boyden chamber) migration assays (26). Furthermore, we previously showed that knockdown of MITF expression in NZM6 and NZM15 melanoma cell lines led to an almost 4-fold increase in the migration rates of the cells (26).

Our results suggest that PAX3 might play a role in melanoma cell invasion (rather than in proliferation), and our data predict that the effect of increased signaling through the PAX3/POU3F2 pathway on cell migration would be most pronounced in melanoma cells *in vivo* where the MITF expression levels are relatively low. In addition, reduced MITF expression levels would occur when POU3F2 expression is elevated, and this would also correspond to instances *in vivo* in melanoma tissues when pigment production is reduced (19). In contrast, in melanoma cells *in vivo* where the MITF expression is high there is likely to be a minimal role of the PAX3-POU3F2 axis in promoting melanoma cell invasion.

We reported previously that PAX3 is extensively expressed in melanocytes, nevi and melanoma tissues (6), and that expression levels of PAX3 and MITF are highly variable in melanoma cell lines, and are not concordant with each other, especially comparing individual melanoma cells in culture (10). We have also previously reported that PAX3 does not transcriptionally activate *MITF* in melanoma cells (10), an observation contrary to that outlined in a number of contemporary melanoma research papers. Current belief has it that in melanoma, PAX3 transcriptionally activates MITF and therefore functions in an epistatic relationship with *MITF*. This is a notion held by many in the melanoma field, primarily because in neural crest development and during melanocyte differentiation PAX3 transcriptionally activates *MITF* [reviewed in Ref. (7, 8)]. Our earlier investigations (10) are, to our knowledge, the only comprehensive investigations systematically addressing this notion, demonstrating that PAX3 does not transcriptionally activate *MITF* in melanoma cells. Further, as suggested in Figure 2, the relative expression levels of PAX3 and MITF are variable in different regions of melanoma tissue, which is not inconsistent with observations of transient changes in pigment production and of POU3F2 expression associated with melanoma dissemination (19). Indeed, amongst several melanoma cell lines that we have examined previously, we observed relatively large fluctuations in MITFm expression, and the variations in PAX3 expression level were not as great as MITFm (10).

Our RNAi data in Figure 4 suggest that PAX3 and MITF expression contribute in distinct ways to cell migration, leading to the suggestion that it is the relative strengths of signals in the PAX3-POU3F2 axis versus the MITF-*miR-211* axis that define the strength of lineage commitment in melanoma cells, and the migratory behavior of the melanoma cells. This latter interpretation could be related to the mechanisms involved in phenotype switching of melanoma cells, where melanoma cells are induced to de-differentiate from a relatively strongly differentiated and proliferative melanoma cell lineage to a more stem cell-like phenotype with reduced proliferation and enhanced cell migration (10, 17, 29).

In conclusion, here we have presented new evidence that PAX3 and MITF expression have independent and opposing effects in melanoma. In line with these data we are proposing a genetic switch theory as a working model to guide future experimental approaches investigating the mechanisms underlying melanoma progression, and the acquisition of resistance and invasiveness. As more work is carried out to test the predictions made from the genetic switch theory, this in turn should lead to a better understanding of mechanisms associated with melanoma progression. Developing a clear description of the mechanisms in melanoma associated with key molecular pathways and phenotype switching is highly likely to be important for the successful treatment of invasive melanoma.

## Materials and Methods

### Human tissues, cell lines, and cell culture

Normal human skin, human nevus, and melanoma tissues, which were formalin-fixed and embedded in paraffin blocks were obtained from Dunedin hospital. Approval for the use of the archival formalin-fixed paraffin-embedded tissues in research was from the New Zealand Multi-Region Ethics Committee. A panel of metastatic human melanoma cell lines, NZM1-NZM48, established in culture from human MM tissue explants (25) were grown at 37°C in a low oxygen (5% O_2_, 5% CO_2_) humidified atmosphere in ITS (Roche, Penzberg, Germany) medium comprising α-modified minimal essential medium (Invitrogen, Carlsbad, CA, USA) insulin (10 μg/mL), transferrin (10 μg/mL), selenite (10 ng/mL), and 10% fetal bovine serum (FBS) as previously described (25). These cell lines were then subsequently cultured in 5% CO_2_ and 95% air humidified atmosphere in ITS DMEM medium and 10% FBS.

### Immunohistochemical and immunofluorescence staining

Tissue sections were cut at 4 μm thickness. Antigen retrieval was performed by heating in 10 mM Tris, 1 mM EDTA buffer, at pH 9, for 30 min. Non-specific antigen reactivity in the sections was blocked by incubation in 1× BSA (ImmSolv LLC, Seattle, DC, USA) for 30 min at room temperature, followed by incubation with PAX3 antibody [Developmental Studies Hybridoma Bank (DSHB, Iowa, IA, USA)] diluted 1:50 or MITF antibody (Invitrogen, Clone C5 + D5) diluted 1:100 in 0.3 × BSA/PBS buffer and incubated at 4°C overnight. The slides were then washed and incubated with horse anti-mouse biotin conjugated antibody and Vectastain Elite ABC kit (Vector Laboratories, Burlingame, CA, USA). DAB was used as the substrate chromagen and hematoxylin as the counterstain. For dual immunofluorescence staining, mouse anti-PAX3 antibody (DSHB) and rabbit anti-MITF antibody (Atlas Antibodies, 1:50) were co-incubated for 2 h at room temperature, then followed by washing, and secondary antibodies (goat anti-mouse – Alexa fluor-568 and goat anti-rabbit-Alexa fluor-488, both from Invitrogen Molecular Probes, 1:1000 dilutions) incubation for 1 h. The β-tubulin antibody (clone E7) was purchased from DSHB, and the secondary antibody was goat anti-mouse – Alexa fluor-568. The cell fixation and staining processes were the same as described previously (10). Negative control incubations using the same secondary antibody, but omitting the primary antibodies were also carried out and showed negative staining as expected. Images were captured with using a Zeiss Axioplan (Germany) microscope, Diagnostic digital camera (Model# 9.4 Slider-6) and Spot software (USA). Fluorescent light source was from EXFO X-Cite 120.

### siRNA transfections

Cells were cultured in 5% CO_2_ and 95% air humidified atmosphere in ITS DMEM medium and 10% FBS prior to and during *PAX3*-siRNA, and *MITF-*siRNA treatments. A reverse transfection technique was used to deliver siRNAs to melanoma cell lines according to the manufacturer’s instructions (Lipofectamine RNAiMAX; Invitrogen, cat. no. 13778-075). Briefly, 1 μl of Lipofectamine RNAiMAX and 6 pmol of siRNA were used for each well of 24-well-plate, in 100 μl of OPTI MEM I media and 500 μl of cells (6 × 10^4^/mL). *PAX3*-siRNA from Ambion (ID#: 215907): sense, GCCGCAUCCUGAGAAGUAAtt; antisense, UUACUUCUCAGGAUGCGGCtg. MITF-siRNA from Ambion (ID#: 3816): sense, GGACAAUCACAACCUGAUUtt; antisense, AAUCAGGUUGUGAUUGUCCtt. An siRNA against Luciferase (from Dharmacon Research Inc.) was used as negative control scramble siRNA, mRNA target sequence AACGUACGCGGAAUACUUCGA.

### Quantitative real-time reverse transcription-PCR analysis

Total RNA was extracted from cell lines and subject to RT-qPCR analysis as previously described (10), with the exception that a Roche Lightcycler was used for amplification and analysis. The primer sequences and the amplicon sizes of the PCR products are shown in Table 1.

**Table 1:** **PCR primers and amplicon sizes**.

Gene		Primer sequence (5′ →3′′)	Amplicon (bp)
*PAX3*	F	ACGCGGTCTGTGATCGAAACA	126
	R	TCTCGCTTTCCTCTGCCTCCTT	
*MITF*	F	GAGCACTGGCCAAAGAGAGG	82
	R	ATGCGGTCATTTATGTTAAATCTTCTTC	
*POU3F2*	F	TTTCCTCAAATGCCCCAAG	108
	R	TTTCTGTCTCCTGTTACAAAACCA	
*GNB2L1*	F	CACAACGGGCACCACCAC	138
	R	CACACACCCAGGGTATTCCAT	

### Transwell migration assays

Transwell migration assays were carried out using 1 × 10^5^ cells seeded into transwell inserts with 8 μm micropore filters (Becton Dickinson) as previously described (26).

## Conflict of Interest Statement

The authors declare that the research was conducted in the absence of any commercial or financial relationships that could be construed as a potential conflict of interest.
